# Firefly luciferase offers superior performance to AkaLuc for tracking the fate of administered cell therapies

**DOI:** 10.1007/s00259-021-05439-4

**Published:** 2021-07-27

**Authors:** Francesco Amadeo, Antonius Plagge, Anitta Chacko, Bettina Wilm, Vivien Hanson, Neill Liptrott, Patricia Murray, Arthur Taylor

**Affiliations:** 1 Cellular Therapies Laboratory, NHS Blood and Transplant, Liverpool, UK; 2 Department of Molecular Physiology and Cell Signalling, University of Liverpool, Liverpool, UK; 3 Centre for Preclinical Imaging, University of Liverpool, Liverpool, UK; 4 Present Address: Department of Cell and Developmental Biology, University College London, London, UK; 5 Department of Pharmacology & Therapeutics, University of Liverpool, Liverpool, UK

**Keywords:** Bioluminescence imaging, Reporter genes, Mesenchymal stromal cells, Cell therapies, Animal models

## Abstract

**Introduction:**

A novel, red-shifted bioluminescence imaging (BLI) system called AkaBLI has been recently developed for cell tracking in preclinical models and to date, limited data is available on how it performs in relation to existing systems.

**Purpose:**

To systematically compare the performance of AkaBLI and the standard Firefly luciferase (FLuc) systems to monitor the biodistribution and fate of cell therapies in rodents.

**Methods:**

Umbilical cord mesenchymal stromal cells (MSCs) were transduced to produce two genetically engineered populations, expressing either AkaLuc or the engineered FLuc luc2. The bioluminescence of AkaLuc^+^ and FLuc^+^ cells was assessed both in vitro (emission spectra, saturation kinetics and light emission per cell) and in vivo (substrate kinetics following intraperitoneal and subcutaneous administration and biodistribution of the cells up to day 7).

**Results:**

Introduction of the reporter genes has no effect on MSC phenotype. For BLI, the FLuc system is superior to AkaBLI in terms of (i) light output, producing a stronger signal after subcutaneous substrate delivery and more consistent signal kinetics when delivered intraperitoneally; (ii) absence of hepatic background; and (iii) safety, where the AkaLuc substrate was associated with a reaction in the skin of the mice in vivo.

**Conclusion:**

We conclude that there is no advantage in using the AkaBLI system to track the biodistribution of systemically administered cell-based regenerative medicine therapies in vivo.

**Supplementary Information:**

The online version contains supplementary material available at 10.1007/s00259-021-05439-4.

## Introduction

Bioluminescence imaging (BLI) is a standard non-invasive technique that can be used to evaluate cell biodistribution, safety and viability longitudinally in small animals [1–4]. BLI is based on the emission of light during the oxidation of a substrate (luciferin) catalysed by a specialised enzyme (luciferase). Cells expressing a luciferase genetic reporter can then be easily detected by BLI in vivo following the administration of the relevant substrate. Firefly luciferase (FLuc) and the substrate D-Luciferin constitute one of the main BLI systems used in vivo in small animals [5–7]. When D-Luciferin is oxidised by FLuc, the wavelength of ~ 50% of the emitted photons is below 600 nm [5, 8] which results in them being strongly attenuated by mammalian tissues [9], reducing sensitivity.

Different approaches have been used to produce BLI systems with near-infrared light emission peaks [5, 10–14]. A recently developed novel BLI system consists of a luciferin analogue, the Akalumine-HCl, and a mutated form of FLuc, called AkaLuc [15, 16]. This BLI system has a light emission peak around 650 nm and has been shown to provide a light output that is 52-fold stronger than that generated by FLuc when used in vivo [15]. This dramatic increase in sensitivity meant it was possible to detect signal from a single cell following intravenous injection [15]. All these characteristics make the AkaLuc/Akalumine-HCl (AkaBLI) system very promising for cell tracking. However, a rigorous comparison of the FLuc/D-luciferin and AkaBLI systems has not yet been undertaken, nor has AkaBLI been validated for applications in regenerative medicine.

Currently, there are over 3300 clinical trials active or in recruitment all over the world (clinicaltrials.gov) where mesenchymal stromal cells (MSCs) are being assessed for their therapeutic potential. Consequently, collecting adequate preclinical data relating to the safety, biodistribution, and fate of these cells is of major interest, and can be achieved in small animals using BLI [17]. Here, we applied genetic engineering to produce two umbilical cord (UC-)MSCs populations, encoding either for the engineered FLuc luc2 or AkaLuc, and we compared these two populations both in vitro and in vivo, to identify which of the two BLI systems is the most suitable for tracking the biodistribution of UC-MSCs in vivo.

## Methods

### Cell isolation and culture

We obtained p3 human umbilical cord-derived mesenchymal stromal cells (UC-MSCs) from the National Health Service Blood and Transplant (NHSBT, UK), and we cultured them following standard mammalian tissue culture protocols. In short, the cells were grown in MEM-α containing GlutaMAX (Gibco) and supplemented with 10% foetal bovine serum (FBS; Sigma) and kept at 37 °C in a humidified incubator, with 5% CO_2_.

### Generation of reporter cell lines

We transduced UC-MSCs with lentiviral vectors encoding either the luc2 firefly luciferase (FLuc) or the AkaLuc reporter. The pHIV-Luc2-ZsGreen vector was a gift from Bryan Welm and Zena Werb (Addgene plasmid #39,196) and the pcDNA3-Venus-AkaLuc vector was a gift from Atsushi Miyawaki (Riken Plasmid #RDB15781) [15]. The pHIV-AkaLuc-ZsGreen vector was created by replacing the luc2 sequence with AkaLuc, generating two vectors that shared the same backbone, promoter (constitutive elongation factor 1-α, EF1α, promoter) and the ZsGreen fluorescent protein downstream of the bioluminescence reporter via an IRES linker, as described in Supplementary Fig. 1a. Lentiviral particles were produced using standard protocols [18] by co-transfection of HEK cells with the transfer vector (pHIV-Luc2-ZsGreen or pHIV-AkaLuc-ZsGreen), an envelope plasmid (pMD2.G) and a packaging plasmid (psPAX2), concentration by ultracentrifugation and titration using HEK cells, based on ZsGreen expression.

We infected p5 UC-MSCs with a multiplicity of infection (MOI) of 5 in the presence of 20 μg/mL protamine sulphate (Sigma). We further employed a spinoculation protocol, where the plate containing the cells and the viral particles was centrifuged at 750 g for 1 h and then incubated at 37 °C for an extra hour, before a washing step with fresh medium [19]. The cells were then grown for another 7 days before they were sorted based on ZsGreen fluorescence, leading to two p6 UC-MSC populations that were 100% positive for either the FLuc or the AkaLuc. After expansion, we cryopreserved the transduced cells at p7. In vitro experiments were carried out with cells at p8-9 and all in vivo bioluminescence experiments with cells at p8.

To determine marker expression via flow cytometry, we detached the cells with Trypsin–EDTA (0.05% trypsin, 0.02% EDTA) and stained them with anti-CD44 (APC, #130–113-893, Miltenyi Biotec), anti-CD45 (APC, #130–113-676, Miltenyi Biotec), anti-CD73 (APC, #130–097-945, Miltenyi Biotec), anti-CD90 (APC, #130–117-534, Miltenyi Biotec), anti-CD105 (APC, #130–099-125, Miltenyi Biotec), IgG1 mouse isotype (APC, #130–113-758, Miltenyi Biotec), or IgG2 mouse isotype (APC, #130–113-831, Miltenyi Biotec) according to the manufacturer’s instructions. An extra vial of each cell population was used as unstained blank. We acquired the data with a FACScalibur (BD Biosciences) and we analysed a minimum of 10^4^ events for each marker.

### Doubling time

From p7 to p12, the cells were counted and plated at 3 × 10^3^ cells/cm^2^ at each passage, and the doubling time calculated using the following equation:

$$Td=\frac{t}{{Log}_{2}(\frac{{N}_{t}}{{N}_{0}})}$$

where Td is the doubling time, N_t_ is the number of cells at time t and N_0_ is the number of cells seeded, from which the number of doublings was calculated based on the time the cells had been in culture.

### Morphological analysis

We seeded the cells at 3 × 10^3^ cells/cm^2^ into 8-well chamber slides (Corning) and allowed them to attach for 16 h. They were then fixed with formaldehyde (4% w/v in PBS, pH 7) for 20 min at room temperature (RT), washed with PBS, permeabilised with 0.1% (v/v) Triton X-100 in PBS and incubated with Alexa Fluor 594 Phalloidin (#A12381, ThermoFisher) [165 nM] in PBS with 1% (w/v) bovine serum albumin (BSA) for 1 h at RT. We used 4′,6-diamidino-2-phenylindole (DAPI) [143 nM] as a counterstaining for the nuclei. We acquired the fluorescence images with a Leica DM2500 microscope coupled to a DFC350 FX camera. Finally, we used ImageJ to manually delineate the cells using images acquired at × 100 magnification and based on the Phalloidin staining. The ImageJ “Analyze Particles” tool was used to determined cells’ area, perimeter and circularity.

### Bioluminescence imaging

All bioluminescence data was obtained with an IVIS Spectrum instrument (Perkin Elmer) and normalised to radiance (photons/second/centimeter^2^/steradian). Where applicable, we used the region of interest (ROI) tool to determine the signal of a specific area (e.g. a single well from a well plate or a single animal).

To determine the light emission spectra of the reporters, we harvested 5 × 10^3^ cells and suspended them in 50 μL of PBS in a 0.2 mL vial. We added the substrates to the vials immediately before data acquisition to a final concentration of 160 μM for Akalumine-HCl (#6555, Bio-Techne®) and 640 μM for D-Luciferin (E1605, Promega). Then, the light emitted from 500 to 840 nm was measured at 20-nm steps by employing the emission filters available in the system.

We measured the substrate saturation in vitro by seeding 1.5 × 10^3^ cells/well into an optical bottom 96-well plate with black walls (#165,305, ThermoFisher). We allowed the cells to attach for 3 h prior to adding the substrate to the medium to a final concentration ranging from 2.5 μM to 5.12 mM, after which data was acquired immediately. We employed a similar protocol to calculate the flux per cell, but a range of cell densities was used (156 to 2 × 10^4^ cells/well) and the substrate concentration kept constant (160 μM Akalumine-HCl or 5.12 mM D-Luciferin). Experiments were repeated 3 times, with a technical triplicate each.

### Animal experiments

We used 7–9-week-old C57 Black 6 (C57BL/6) albino female mice for all animal experiments. Mice were obtained from a colony managed by the Biomedical Services Unit at the University of Liverpool (UK) which had been established from the B6N-*Tyr*^*c−Brd*^/BrdCrCrl strain originally purchased from Charles River (Italy). Mice were housed in individually ventilated cages (IVCs) under a 12-h light/dark cycle and provided with standard food and water ad libitum. All animal procedures were performed under a licence granted under the Animals (Scientific Procedures) Act 1986 and were approved by the University of Liverpool Animal Ethics Committee.

For cell administration, the mice were anaesthetised with isoflurane and intravenously (IV) injected with 2.5 × 10^5^ UC-MSCs suspended in 100 μL of PBS. Then, under the same anaesthesia session, the animals received a subcutaneous (SC) or intraperitoneal (IP) injection of the substrate prior to imaging with the IVIS. All imaging procedures in subsequent days were carried out under anaesthesia, including the administration of the substrates. For kinetic analysis of the signal, BLI data was acquired continuously from substrate administration for up to 30 min.

The AkaLuc-HCl substrate was used at a fixed dose of 100 μL of a 30 mM stock, which was previously reported to give a maximum signal in vivo [15]. D-Luciferin was used at either 10 μL/g of a 47 mM stock solution (“low dose”) or 20 μL/g of a 144.5 mM stock solution (“high dose”). To keep the administered volumes constant between groups that received D-Luciferin, animals that received the “low dose” were injected with an additional 10 μL/g of PBS, irrespective of the administration route. Quantification of BLI signal from animals was obtained from a region of interest covering the whole body of an individual mouse. More information about the experimental conditions for each animal experiment can be found in Supplementary Tables 1–3. Our data is reported in line with the ARRIVE guidelines [20].

### Statistical analysis

All values in graphs are represented as mean ± standard deviation. The statistical analysis was performed using the GraphPad software. The type of statistical test and the number of replicates included in the analyses are indicated in the figure legends.

## Results

### Impact of reporter gene expression on MSC properties

**Fig. 1:**
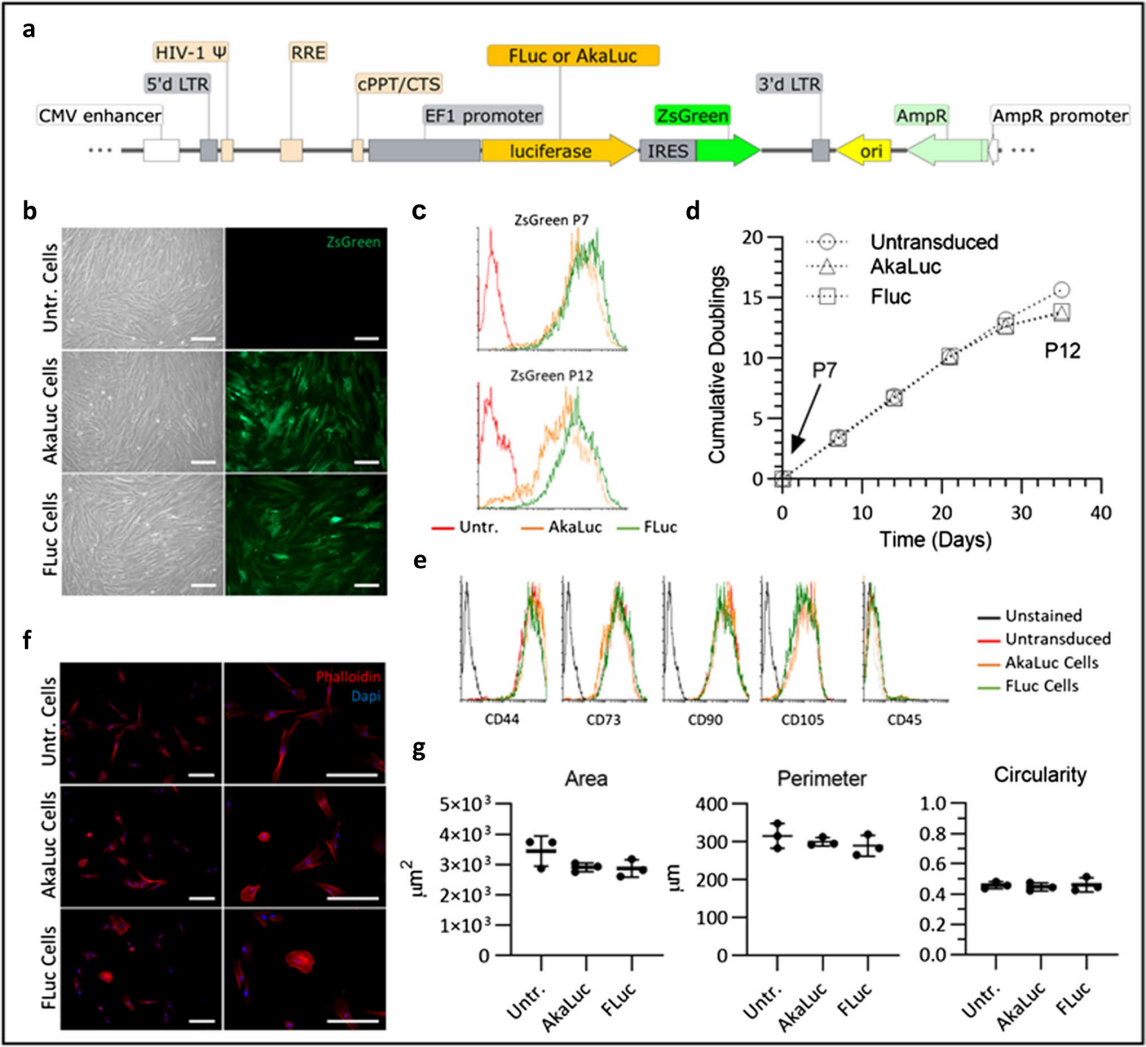
AkaLuc and FLuc transduced cells retain characteristics of untransduced UC-MSCs. **a** Schematic of the lentiviral vector used to generate MSCs expressing the reporters. The same vector backbone was used, and only the gene encoding for the luciferase was modified for expression of either FLuc or AkaLuc. Image produced using SnapGene software (from Insightful Science; available at snapgene.com). **b** Representative phase contrast and fluorescence images of untransduced, AkaLuc and FLuc expressing MSCs when fully confluent. ZsGreen expression can be observed in the green channel for the transduced cells. Scale bar = 200 μm. **c** Levels of transgene expression in AkaLuc^+^ and FLuc^+^ MSCs at different passages. ZsGreen expression was measured via flow cytometry at p7 and p12 and untransduced MSCs were used as a negative control. The levels of ZsGreen are equivalent for both populations at p7 and remain largely unchanged up to p12, with a slight reduction for AkaLuc cells. The mean fluorescence intensity (MFI) of FLuc population shifted from 1023 at p7 to 708 at p12, while the MFI of AkaLuc population shifted from 746 at p7 to 388 at p12 (arbitrary units). **d** Cumulative doubling measured from passages 7 to 12 shows that cells proliferate at the same rate up to p11. **e** Flow cytometry analysis of markers that identify MSCs shows that all populations are positive for CD44, CD73, CD90 and CD103, and negative for CD45. **f** Fluorescence images of cells stained with phalloidin (f-actin, red) and DAPI (nuclei, blue), acquired at × 100 (left panel) and × 200 (right panel) magnification. Scale bar = 200 μm. **g** Area, perimeter, and circularity, as measured based on the phalloidin staining of at least 50 cells per replicate, show that MSCs retain their morphology after transduction with either of the reporters. Data are displayed as mean ± SD from *n* = 3. One-way ANOVA revealed no statistically significant difference between the three cell populations

We produced UC-MSCs expressing FLuc or AkaLuc by lentiviral transduction with vectors sharing the same backbone and encoding for either of the reporters (Fig. 1a). Figure 1b shows phase contrast images of confluent control, AkaLuc^+^ and FLuc^+^ MSCs, and the respective green fluorescence images, showing ZsGreen expression by all cells. This was also confirmed by flow cytometry, with both cell populations having close mean green fluorescence intensities, indicating similar levels of expression of the transgenes (Fig. 1c). Cumulative doubling of the genetically modified cells from p7 to p10 (Fig. 1d) was the same as the untransduced control, suggesting that proliferation rate was not affected by insertion of the reporters. After this passage, all populations appear to display a slight decrease in doublings, which is more pronounced for the transduced cells (Fig. 1d). Flow cytometry analyses of ZsGreen expression at p7 and p12 show that FLuc^+^ cells are still all positive at p12, while there was a little reduction in the level of expression in AkaLuc^+^ cells (Fig. 1c). Expression of common mesenchymal markers was similar in all three populations, with a good overlap of the flow cytometry data (Fig. 1e), revealing that the marker expression was not altered by either of the reporter genes and that all cells were negative for CD45 and positive for CD44, CD73, CD90, and CD105 (Fig. 1e). Morphological analysis of the three cell populations showed no statistically significant difference in the area, perimeter, or circularity of MSCs, regardless of the genetic modification (Fig. 1f and g).

### Characterisation of the reporter systems in vitro

**Fig. 2:**
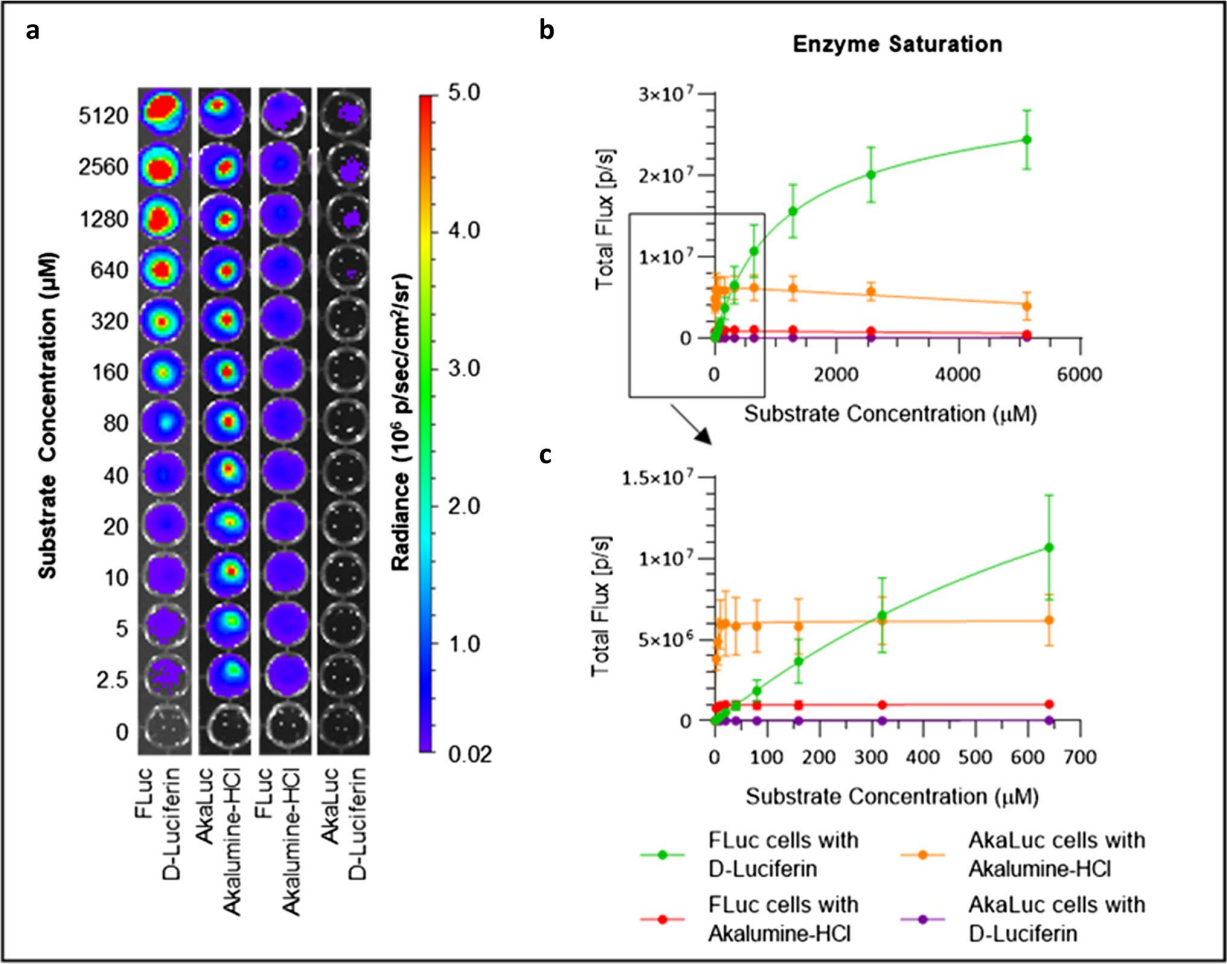
The AkaLuc and FLuc reporters saturate at different substrate concentrations in vitro. AkaLuc and FLuc expressing MSCs were seeded at a density of 1.5 × 10^3^ cells/well and treated with increasing concentrations of Akalumine-HCl or D-Luciferin (2.5 μM to 5.12 mM). **a** Representative images of a well plate immediately after the substrate addition to the cells. **b**, **c** Light output (flux) as a function of substrate concentration, where **b** shows the signal obtained from 2.5 μM to 5.12 mM and **c** from 2.5 μM to 640 μM. Data are displayed as mean ± SD from *n* = 3. Acquisition parameters: no emission filters, 13.3 cm field of view (FOV), f-stop of 1, binning of 8 and 10 s of exposure

Imaging of MSCs with increasing concentrations of Akalumine-HCl or D-Luciferin revealed that FLuc^+^ cells display significant light emission with either of the substrates, while AkaLuc^+^ cells only emit significant light when exposed to Akalumine-HCl (Fig. 2a). Quantification of the signal in individual wells shows that both FLuc^+^ and AkaLuc^+^ cells in the presence of Akalumine-HCl display maximum flux at very low substrate concentrations, while FLuc cells incubated with D-Luciferin display a slow increase in signal up to 5.12 mM (Fig. 2b, c). Both cell populations are saturated and reach a plateau at approximately 20 μM for Akalumine-HCl (Fig. 2c, Supplementary Fig. 2a). At this concentration, the light emitted by AkaLuc^+^ cells is 11.8- and sixfold greater than the signal emitted by FLuc^+^ cells with D-Luciferin or Akalumine-HCl, respectively (Fig. 2c). Interestingly, the signal from AkaLuc and FLuc cells exposed to Akalumine-HCl at concentrations higher than 640 μM drops slightly (Fig. 2b, Supplementary Fig. 2b). Even when increasing the dose of the substrates to up to 5.12 mM, the signal generated by FLuc^+^ cells in the presence of D-Luciferin does not fully saturate, although the slope of the curve starts to level out. At this concentration, the signal produced by FLuc^+^ cells is fourfold greater than the signal generated by AkaLuc^+^ cells saturated with Akalumine-HCl (Fig. 2b).

**Fig. 3:**
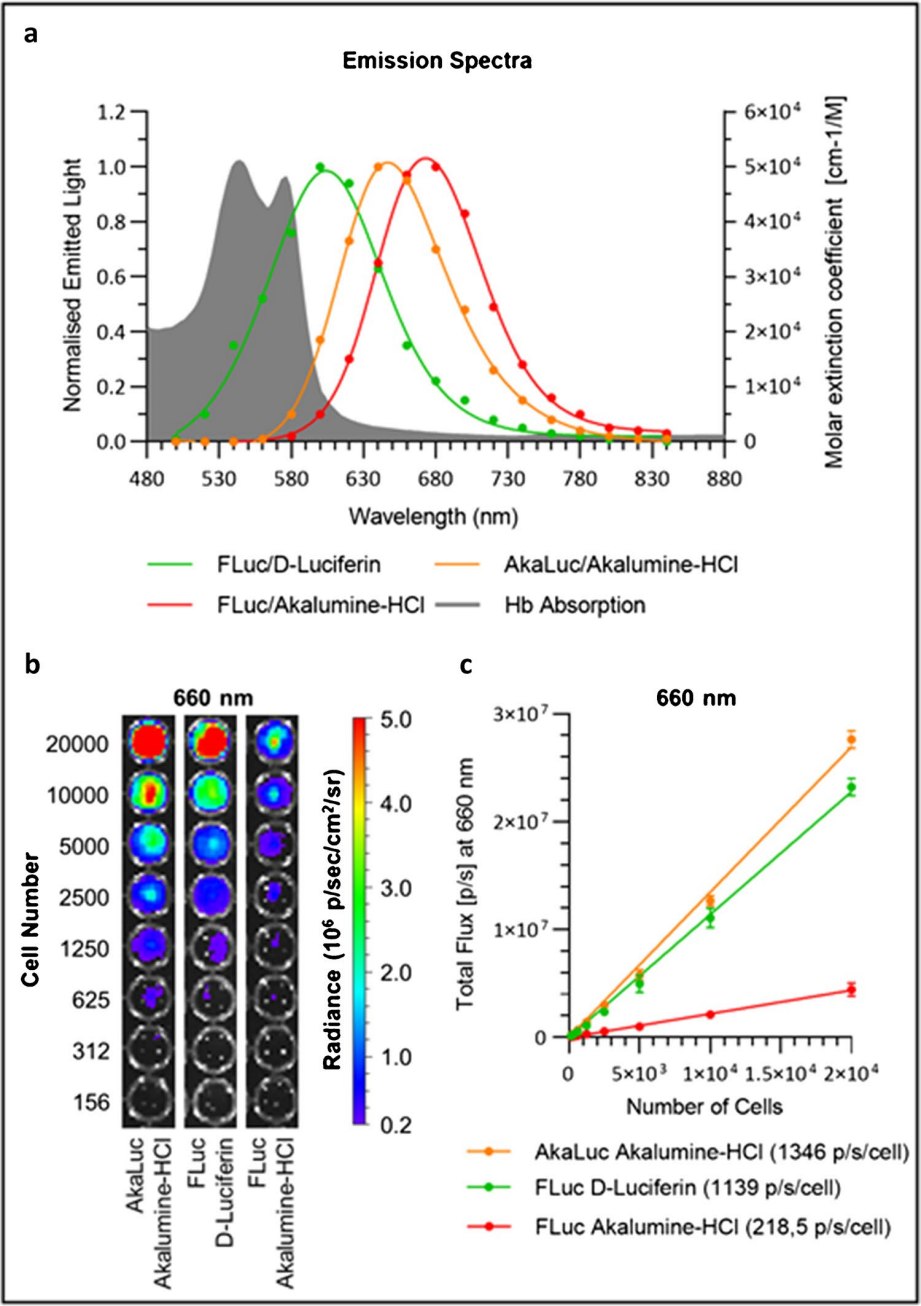
Emission spectra and light output as a function of cell density for the AkaLuc and FLuc reporter systems. **a** Emission spectrum for each enzyme/substrate pair, as measured by acquiring the signal emitted from 500 to 840 nm at 20 nm steps. Data is normalised to the peak value of each condition. The AkaLuc/Akalumine-HCl system displays a peak at 650 nm, whereas FLuc cells display a peak at 600 nm in the presence of D-Luciferin and 670 nm in the presence of Akalumine-HCl. Haemoglobin (Hb) absorption (average of values of oxy-Hb and deoxy-Hb) is plotted in grey (unit of measure: molar extinction coefficient as shown on the right axis). **b**, **c** AkaLuc and FLuc expressing MSCs were seeded at a density of 156 to 2 × 10^4^ cells/well and treated with a saturating concentration of the substrates (160 μM Akalumine-HCl or 5.12 mM D-Luciferin). AkaLuc expressing cells were treated with Akalumine-HCl only, whereas FLuc expressing cells were treated with AkaLuc-HCl or D-Luciferin. The signal was acquired using a 660 nm filter. **b** Representative images of a well plate immediately after the substrate addition. **c** Light output (flux) as a function of cell concentration, with linear regression curves. The slope of each curve represents the flux/cell and is shown in the legend. Data are displayed as mean ± SD from *n* = 3. Acquisition parameters: 13.3 cm FOV, f-stop of 1, binning of 8 and 10 s of exposure

Measurements of the spectral properties of the reporters show that AkaLuc^+^ cells display a peak at 650 nm when supplied with Akalumine-HCl. FLuc^+^ cells, on the other hand, display a peak at 670 nm when supplied with Akalumine-HCl and at 600 nm when supplied with D-Luciferin (Fig. 3a). While a significant portion of the light emitted by the FLuc/D-Luciferin system overlaps with the absorption of haemoglobin, almost all the light emitted by the AkaLuc/Akalumine-HCl does not (Fig. 3a).

To obtain the flux per cell, we plated MSCs at various densities from 156 to 2 × 10^4^ cells/well and we subsequently imaged them in the presence of Akalumine-HCl or D-Luciferin at a final concentration of 160 μM and 5.12 mM, respectively. This corresponds to concentrations well above the saturation for AkaLuc/Akalumine-HCl and the highest dose of D-Luciferin used in this study, which provided the strongest signal with FLuc. We measured the light output with a 660-nm filter (19.57 nm bandwidth), which is physiologically relevant as it is in the near-infrared optical imaging window and also matches the emission peak of the AkaLuc/Akalumine-HCl system (650 nm). Both reporter systems display considerable light emission (Fig. 3b) and a linear regression of the signal measured in each well demonstrates that the flux of AkaLuc^+^ cells (1346 p/s/cell) is nearly sixfold higher than FLuc^+^ cells with Akalumine-HCl (218.5 p/s/cell), but only slightly stronger than FLuc^+^ cells with D-Luciferin (1139 p/s/cell) (Fig. 3c). If no light emission filter is applied and data is acquired for the whole spectrum, the light output from FLuc/D-Luciferin significantly outperforms AkaLuc/Akalumine-HCl (Supplementary Fig. 3).

### Light emission kinetics in vivo

**Fig. 4:**
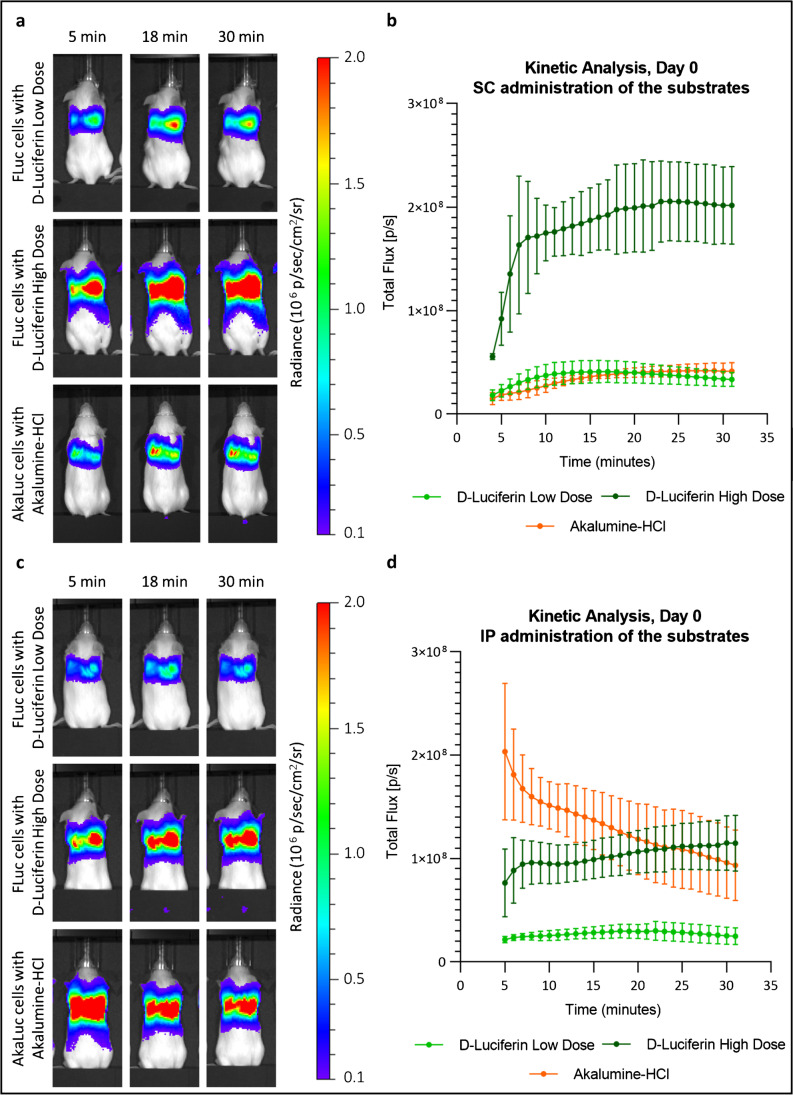
FLuc and AkaLuc systems display similar signal kinetics in vivo when the substrate is administered subcutaneously, but not when administered intraperitonially. MSCs (2.5 × 10^5^) expressing either AkaLuc or FLuc were administered via the tail vein. The mice then received the substrates either subcutaneously or intraperitonially, under the same anaesthesia session, after which they were imaged every minute for 30 min (kinetic analysis). **a** Representative images of the mice 5, 18 and 30 min post -SC administration of the substrates (radiance scale from 1 × 10^5^ to 2 × 10^6^ p/s/cm^2^/sr). **b** Light output (flux) as a function of time, from minute 4 to minute 31. Data are displayed as mean ± SD from *n* = 3. **c** Representative images of the mice 5, 18, and 30 min post-IP administration of the substrate (radiance scale from 1 × 10^5^ to 2 × 10^6^ p/s/cm^2^/sr). **d** Light output (flux) as a function of time, from minute 5 to 31. Data are displayed as mean ± SD from *n* = 4. Low-dose D-Luciferin = 0.47 mmol/kg; high dose D-Luciferin = 2.89 mmol/kg; Akalumine HCl = 100 μL of 30 mM solution. Acquisition parameters: no emission filter, 22.8 cm FOV, f-stop of 1, binning of 8 and a maximum exposure of 45 s for each time point

To determine the kinetic of the BLI signal in vivo, mice that received 250 × 10^3^ FLuc^+^ or AkaLuc^+^ MSCs were imaged every minute for 30 min after administration of the substrates. From this point on, we compare only the FLuc/D-Luciferin and AkaLuc/Akalumine-HCl systems and we investigate two doses of D-Luciferin: the commonly reported 0.47 mmol/kg and a higher dose of 2.89 mmol/kg. We used Akalumine-HCl at the fixed dose of 100 μL of a 30-mM solution, which has previously been reported to be associated with maximum signal in vivo [15]. Representative bioluminescence images of mice acquired 5, 18, and 30 min post-SC or IP injection of the substrates are shown in Fig. 4a, c and are displayed in the same colour scale to allow a direct comparison of the data. A signal is detected from all mice irrespective of the substrate administration route, which shows cells lodging in the lungs (Fig. 4a, c). In the SC route, the signal reaches a maximum between 16 and 28 min after the administration of the substrate (Fig. 4b). The signal from FLuc^+^ cells stays stable for about 7 min before starting to decrease. The light emitted by AkaLuc^+^ cells, on the other hand, seems to remain stable after reaching its maximum (Fig. 4b). For both reporters, a window exists at 20 min post administration of the substrate where over 96% of maximum light output is achieved, which we have used for subsequent experiments. Data acquisition 24 h post cell administration confirmed this time course (Supplementary Fig. 4). In the IP route, the FLuc system displays a time course that resembles that obtained following SC administration, with a rapid increase of the signal in the first minutes post administration, followed by a slower increase up to minute 30 (Fig. 4d). By contrast, the AkaLuc system resulted in maximum signal intensity shortly after the injection of the substrate (min 5, Fig. 4b) followed by a rapid drop in the signal. Similar analysis performed on day 1, day 3, and day 7 shows that while the FLuc system displays the same kinetics at all time points, the AkaLuc system changed from day 3, with an increase in flux over time (Supplementary Fig. 5).

### Reporter gene sensitivity and specificity in vivo

**Fig. 5:**
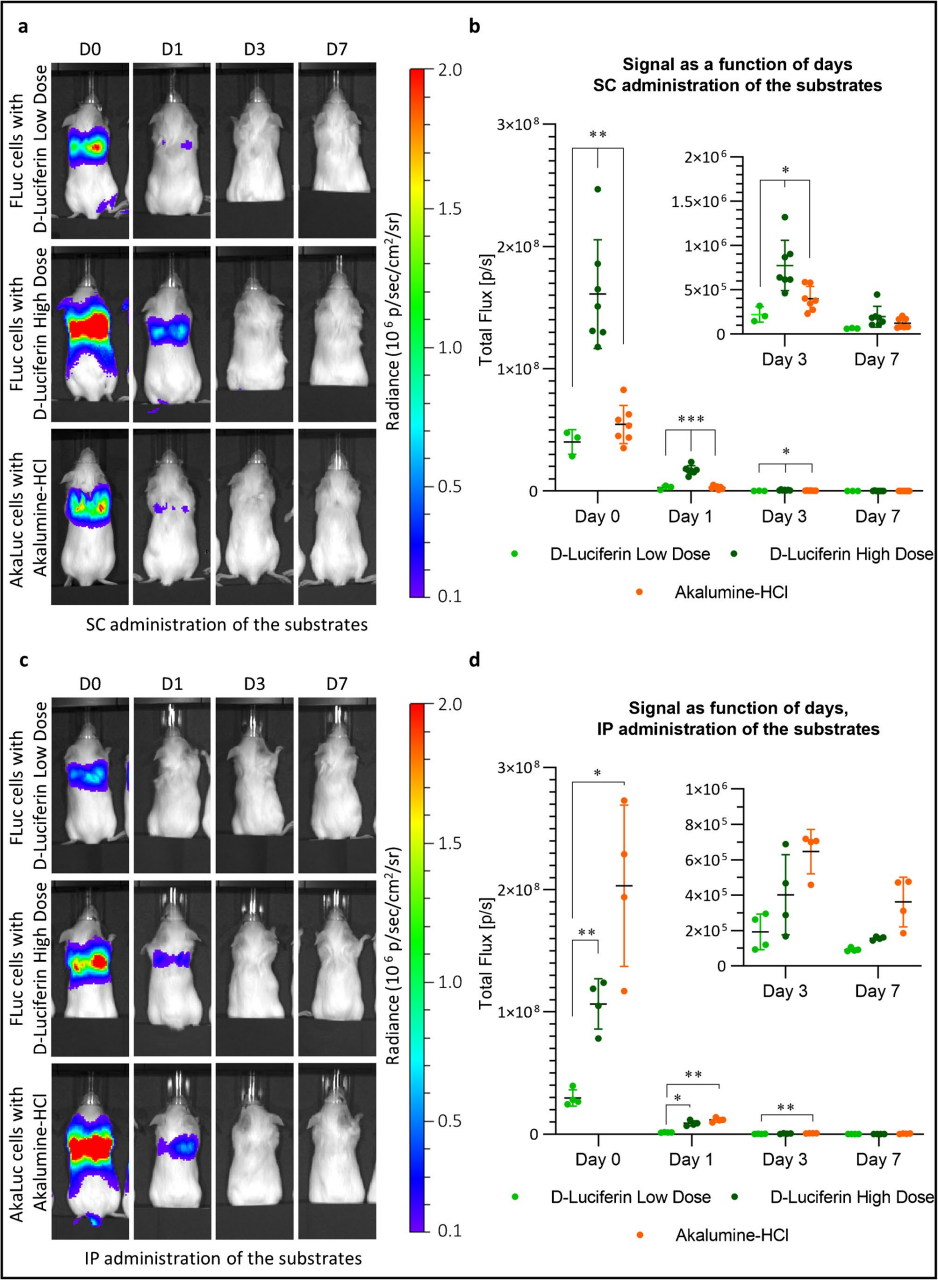
The AkaLuc and the FLuc reporter systems display a different sensitivity over time following SC and IP administration of the substrates. UC-MSCs (2.5 × 10^5^) expressing either the FLuc or the AkaLuc transgene were administered via the tail vein and the mice were imaged at day 0 (administration day) and 1, 3, or 7 days post cell administration. D-Luciferin (low or high dose) or Akalumine-HCl were used as substrates and administered either SC or IP. **a** Representative images of the mice as acquired 20 min post-SC administration of the substrates (radiance scale from 1 × 10^5^ to 2 × 10^6^ p/s/cm^2^/sr). **b** Light output (flux) as a function of time (day). Data are displayed as mean ± SD from *n* = 3 (D-Luciferin low dose), *n* = 7 (D-Luciferin high dose and Akalumine-HCl). The FLuc reporter in combination with a high dose of D-luciferin yields a stronger signal in all days. **c** Representative images of the mice acquired at peak signal of each condition (20 min for D-Luciferin and 5 min (for D0) or 4 min (for D1, 3, and 7) for Akalumine-HCl) following IP administration of the substrates (radiance scale from 1 × 10^5^ to 2 × 10^6^ p/s/cm^2^/sr). **d** Light output (flux) as a function of time (days). Data are displayed as mean ± SD from *n* = 4. Statistical analysis was performed using a two-way ANOVA and Tukey’s multiple comparison post hoc test. *p < 0.05*;* **p < 0.01., ***p < 0.001. Acquisition parameters: no emission filter, 22.8 cm FOV, f-stop of 1 and a binning of 8. FLuc/D-Luciferin exposure time: 45 s for D0 and D1, and 180 s for D3 and D7. AkaLuc/Akalumine-HCl exposure time: 45 s for D0 and 180 s for D1, 3, and 7

To assess the sensitivity of the two reporter genes in vivo, we measured the signal from the MSCs for up 7 days post administration into mice. Based on the data presented in the previous section, all measurements are at 20 min post administration of the substrate except for Akalumine-HCl administered IP, where measurements are at minute 5 for day 0 and at minute 3 for days 1, 3, and 7. In all conditions, there is a strong signal in the lungs on the administration day, which drops by day 1 and is mostly lost by day 3 (Fig. 5a and c). The same data at a lower colour scale are shown in Supplementary Fig. 6, where it is still possible to detect a signal on day 3 in mice injected with FLuc^+^ cells and a high dose of D-Luciferin and in mice injected with AkaLuc^+^ cells and Akalumine-HCl.

The quantification of the signal revealed that when the substrates are administered SC, the signal drops substantially from day 0 to 1 in all conditions (18-fold for AkaLuc/Akalumine-HCl, 13-fold for FLuc/D-Luciferin low dose and ninefold for FLuc/D-Luciferin high dose) and drops again from day 1 to 3 (sevenfold for AkaLuc/Akalumine-HCl, 13-fold for FLuc/D-luciferin low dose and 22-fold for FLuc/D-luciferin high dose) (Fig. 5b). With the IP route, the signal drops from day 0 to 1 in all conditions (17-fold for AkaLuc/Akalumine-HCl, 21-fold for FLuc/D-Luciferin low dose and 12-fold for FLuc/D-Luciferin high dose) and again from day 1 to 3 (18-fold for AkaLuc/Akalumine-HCl, sevenfold for FLuc/D-Luciferin low dose and 22-fold for FLuc/D-luciferin high dose) (Fig. 5d). This drop in signal over time is expected given that it is widely reported that most MSCs die shortly after IV administration [3, 21, 22].

With the SC route, mice injected with FLuc^+^ cells and the high dose of D-luciferin displayed the highest signal output, with the average light emission being 4.5-fold higher than the signal obtained with FLuc cells with low dose of D-Luciferin, and 3.2-fold higher than the signal acquired with AkaLuc cells. The signal emitted by the FLuc system at a low dose of D-luciferin is comparable, and not statistically different to the signal emitted by the AkaLuc system.

With substrate administration IP, the AkaLuc system displays the highest signal output, with the average light emission being 1.6-fold higher than the signal obtained with FLuc^+^ cells with a high dose of D-Luciferin, and 6.1-fold higher than FLuc^+^ cells with a low dose of D-Luciferin (Fig. 5d). However, visualisation of the data at a lower colour scale reveals that mice receiving Akalumine-HCl IP displayed a signal away from the lungs on days 3 and 7 (Supplementary Fig. 6). At 20 min post administration of the substrate, the AkaLuc signal was even stronger and originated from a region corresponding to the liver. This was absent in animals receiving D-Luciferin (Supplementary Fig. 7).

**Fig. 6:**
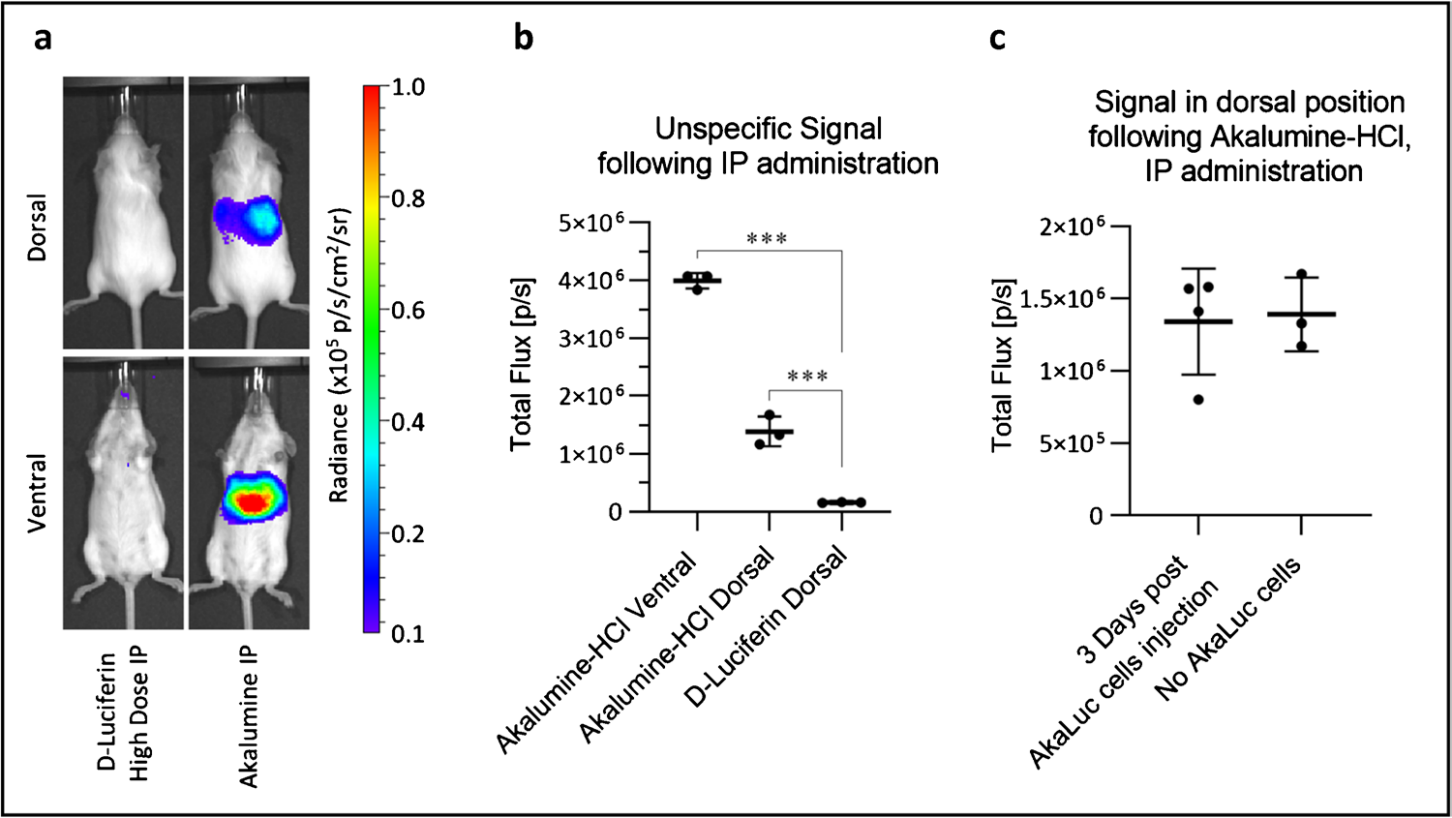
In the absence of cells, Akalumine-HCl generates a non-specific signal from the liver when administered IP. High-dose D-Luciferin or Akalumine-HCl was injected IP in vivo. The mice were then imaged in dorsal (23 min post substrate administration) and ventral (20 min post administration) positions, with no emission filter, a 22.8 field of view, a f-stop of 1, a binning of 8 and an exposure time of 180 s. **a** Representative images of the mice following substrate administration (radiance scale from 1 × 10^4^ to 1 × 10^5^ p/s/cm^2^/sr). **b** Quantification of the non-specific signal detected in the liver region of the mice injected with Akalumine-HCl IP, analysed both in ventral and dorsal position, compared with the signal coming from the liver region of mice injected with high dose D-Luciferin IP. Data are displayed as mean ± SD from *n* = 3. Statistical analysis performed using a one-way ANOVA and Tukey’s multiple comparison post hoc test. ***p < 0.001 post substrate administration. **c** Comparison of the signal detected in the liver of mice injected with Akalumine-HCl, three days post administration of 2.5 × 10^5^ AkaLuc expressing cells IV (*n* = 4) *vs.* the non-specific luminescence from naive mice that received Akalumine-HCl alone (*n* = 3), 20 min. There is no statistically significant difference between the two groups (unpaired t-test). Data are displayed as mean ± SD

To investigate the origin of this signal, we injected naïve mice that did not receive any cells, with the substrates IP. While mice that received luciferin displayed no bioluminescence, those injected with Akalumine-HCl presented a signal coming from the liver which is detectable both in dorsal and ventral positions (Fig. 6a). Quantitative analysis of the data reveals a statistically significant increase in the total flux emitted by Akalumine-HCl alone, both ventrally and dorsally, when compared to D-Luciferin that displays no specific signal (Fig. 6b), which is consistent with a previously observed hepatic background [23]. Importantly, the signal detected in the dorsal position following IP administration of Akalumine-HCl in naïve mice is comparable to the signal detected in mice injected with AkaLuc cells 3 days post administration of the cells (Fig. 6c).

Finally, we identified a reaction in the skin of mice injected SC with Akalumine-HCl. Analysis of the carcasses of mice that were culled on day 10 post administration of the cells, after they had undergone 4 imaging sessions, revealed that animals that had received Akalumine-HCl SC showed lesions that were noticeable when the skin was shaved. These lesions corresponded to the sites of the SC injections and displayed different grades of severity (Supplementary Fig. 8a). No changes to the appearance of the skin of mice treated with D-Luciferin even when using the high dose (Supplementary Fig. 8b) were seen. No abnormalities were identified in the peritoneum of mice that received Akalumine-HCl IP.

## Discussion

The purpose of this study was to determine whether the AkaBLI system truly offers a gain in sensitivity when compared to the well-established FLuc/Luciferin system, with a focus on its use to track the biodistribution of MSCs. Our first goal was to ensure that the expression of the transgene was equivalent between the cell populations we compared. We achieved that by lentiviral transduction using the same MOI and vector, with the only change being the gene encoding for the bioluminescence enzyme, and flow cytometry selection based on equivalent fluorescence intensity of the ZsGreen marker. This is important because the previously reported study on the use of AkaBLI not only involved transfection, a method which only generates transient expression, but also the selection of “highly fluorescent transfectants” [15], meaning that different populations of cells could have differing levels of the transgenes. As measured indirectly by ZsGreen fluorescence, both AkaLuc^+^ and FLuc^+^ MSCs in our study had a comparable and stable expression of the inserts.

Transduction with the vectors did not result in any changes in marker expression, proliferation, or morphology of UC-MSCs, providing evidence that the genetic modification with either luciferase is likely safe. The functionality of the reporters was verified by their specificity to the correct substrates and the emission spectra, with the AkaLuc system displaying a distinct shift to 650 nm, as opposed to the FLuc system which has a maximum emission at 600 nm.

Substrate saturation in vitro, as measured by the concentration required to obtain maximum light output, differed between the two populations. Whereas AkaLuc^+^ cells resulted in a quick saturation of the signal at 20 μM of Akalumine-HCl, FLuc^+^ cells did not fully saturate with luciferin concentrations of up to 5.12 mM. Interestingly, previous comparisons of these systems employed relatively low concentrations of substrates. For example, Kuchimaru and colleagues presented light output data with substrate concentrations of up to 250 μM [16], whereas Iwano and colleagues used substrates at up to 500 μM [15]. These studies suggested that AkaLuc performs better at low substrate concentrations, which is in agreement with our data and studies that have shown a low Km for this and other engineered substrates [24]. However, our observations demonstrate that FLuc is more sensitive when high concentrations of the D-Luciferin substrate are employed. Because D-luciferin substrate has relatively high aqueous solubility and a low cost, we propose that this places D-Luciferin substrate at an advantage in BLI applications when compared to Akalumine-HCl. Even when the light output per cell was measured with a 660-nm filter, which results in a bias towards the AkaLuc system, our data suggest that the two systems are quite similar in terms of light emitted per cell, with 1346 p/s/cell and 1139 p/s/cell for AkaLuc^+^ and FLuc^+^ cells, respectively.

A critical aspect of our study was to determine whether the in vitro data is translated in vivo. This is important because the light output is dependent, among other factors, on the bioavailability of the substrate in the model organism. The standard dose of D-Luciferin used for in vivo BLI is 0.47 mmol/kg body weight [7, 12, 25, 26]. However, it has been shown that higher doses are associated with stronger FLuc signal in vivo [7]. Because of that, we increased the dose of D-Luciferin to 2.89 mmol/kg body weight (approximately sixfold). We achieved that by preparing stock solutions of the substrate at its solubility limit (144.5 mM), followed by increasing the injection volume to up to 20 mL/kg body weight, which is recognised as the maximum volume permitted under animal welfare guidance [27]. Akalumine-HCl was used at the previously reported optimal dose [15]. Both systems were able to show that MSCs are delivered to the lungs after IV administration, regardless of the substrate administration route.

However, there were clear differences between the signal intensity of the AkaLuc and FLuc systems depending on the route of substrate administration: in the SC route, the signal intensity of AkaLuc^+^ cells was not stronger than FLuc^+^ when standard substrate doses were used (100 μL of 30 mM Akalumine-HCl and 0.47 mmol/kg body weight D-Luciferin). By increasing the D-Luciferin dose to 2.89 mmol/kg body weight, the light output obtained with the FLuc system increased significantly by at least threefold. Both BLI systems displayed a similar substrate biodistribution kinetics, with the signal reaching a plateau that allows consistent acquisition of data.

In contrast, when the substrates were administered IP, the signal intensity of AkaLuc^+^ cells was stronger than FLuc^+^ cells, irrespective of whether a high or low dose of luciferin was administered. The AkaLuc^+^ cells displayed a kinetics characterised by a peak immediately after administration of the substrate. This means that for maximum signal intensity, data acquisition needs to proceed quickly after the administration of the substrate. Moreover, due to the rapid decay in signal, the experimental design needs to take into consideration a need for consistent timing between injection of the substrate and data acquisition for each animal. Of note, the kinetics in our study is very different to Iwano’s study [15] involving intracranial administration of cells, where the signal after IP administration of substrate peaked at approximately 15 min. This discrepancy raises further questions on the reliability of the system for imaging cells that might be present in different organs. Furthermore, it is well known that IP administration of BLI substrates can lead to unreliable data due to injection failure rate and irregular distribution of the substrate [28].

In all conditions, regardless of the route of administration of the substrates, the signal dropped from day 0 to 1 and almost disappeared by day 3. This result is consistent with cell death and widely reported in the literature [3, 21, 22]. However, it is important to emphasise that only FLuc in combination with a high dose of D-Luciferin allowed us to unambiguously ascertain that there were still viable cells in the lungs on day 3 post administration.

An additional drawback observed with the AkaBLI system was the existence of an unspecific liver signal when the substrate was injected IP, found even in naïve mice. Although we are not the first to report the accumulation of Akalumine-HCl in the liver [29], or its unspecific bioluminescence [23], what is striking is that on day 3 post cell administration, quantification of the signal shows that the unspecific signal from Akalumine-HCl is actually as strong as the MSC-specific signal from FLuc combined with D-Luciferin, highlighting the potential for serious inaccuracies.

Akalumine-HCl has been reported to be more cytotoxic than most other substrates in vitro [6, 30] and to our knowledge, toxicity has not been previously reported in animals. However, we observed the formation of lesions in sites of SC injection of Akalumine-HCl, with different degrees of severity. We have not investigated the nature of these lesions; however, it is clear that it leads to an abnormal reaction in the animal’s skin. A recent study has suggested that the pH of Akalumine-HCl stock solution is 2.25 [31], which could possibly explain this reaction. Administration of substrate IP has not revealed any abnormalities; this could be because the effects are less severe than SC, because the less targeted nature of IP delivery makes it more difficult to identify the specific injection area, or because SC administration is associated with a higher local concentration of the substrate compared to IP.

In conclusion, our study emphasises the superiority of the FLuc/D-Luciferin system for tracking cells in vivo. In particular, we found that the FLuc system has a better performance in terms of (i) stronger signal with SC administration of substrate, which has no bias towards organs of the torso; (ii) signal kinetics, exhibiting a plateau whether the substrate is administered SC or IP, offering a uniform data acquisition window; and (iii) safety, with a non-toxic substrate that is specific to the enzyme of interest. AkaBLI with IP administration of the substrate might lead to a stronger signal in organs in or close to the peritoneum immediately after substrate administration, but at the expense of the data being potentially inconsistent due to its other limitations. We therefore urge caution when interpreting the data obtained with AkaBLI, suggest that FLuc with a high dose of luciferase ought to be considered as an option for studies that require high sensitivity, and emphasise the need for further systematic comparative studies to determine the performance of more recently developed substrates such as SemPai, iLH2 and NIRLuc2 [24].

## Supplementary Information

Below is the link to the electronic supplementary material.

Supplementary file1 (DOCX 12877 KB)

## Data Availability

All datasets from this study are publicly available on Zenodo, https://doi.org/10.5281/zenodo.4751523
