# Supplementary material for: Firefly luciferase offers superior performance to AkaLuc for tracking the fate of administered cell therapies
Source: Eur J Nucl Med Mol Imaging. 2021 Jul 27;49(3):796–808. doi: 10.1007/s00259-021-05439-4 (PMC8803776; doi:10.1007/s00259-021-05439-4)
Supplement: Supplementary file 1 — Supplementary file1 (DOCX 12877 KB) [file 259_2021_5439_MOESM1_ESM.docx]

**Firefly Luciferase offers superior performance to AkaLuc for tracking the fate of administered cell therapies**

Francesco Amadeo^1,2,3^, Antonius Plagge^2,3^, Anitta Chacko^2^, Bettina Wilm^2,3^, Vivien Hanson^1^, Neill Liptrott^4^, Patricia Murray^2,3^, Arthur Taylor^2,3^

1. Cellular Therapies Laboratory, NHS Blood and Transplant, Liverpool, UK.

2. Department of Molecular Physiology and Cell Signalling, University of Liverpool, Liverpool, UK.

3. Centre for Preclinical Imaging, University of Liverpool, Liverpool, UK.

4. Department of Pharmacology & Therapeutics, University of Liverpool, Liverpool, UK.

Corresponding author e-mail address: [taylora@liverpool.ac.uk](mailto:taylora@liverpool.ac.uk)

Orcid IDs: Francesco Amadeo 0000-0002-3868-2348; Neill Liptrott 0000-0002-5980-8966; Antonius Plagge 0000-0001-6592-1343; Bettina Wilm 0000-0002-9245-993X; Patricia Murray 0000-0003-1316-148X; Arthur Taylor 0000-0003-2028-6694

**Supplemental Information:**


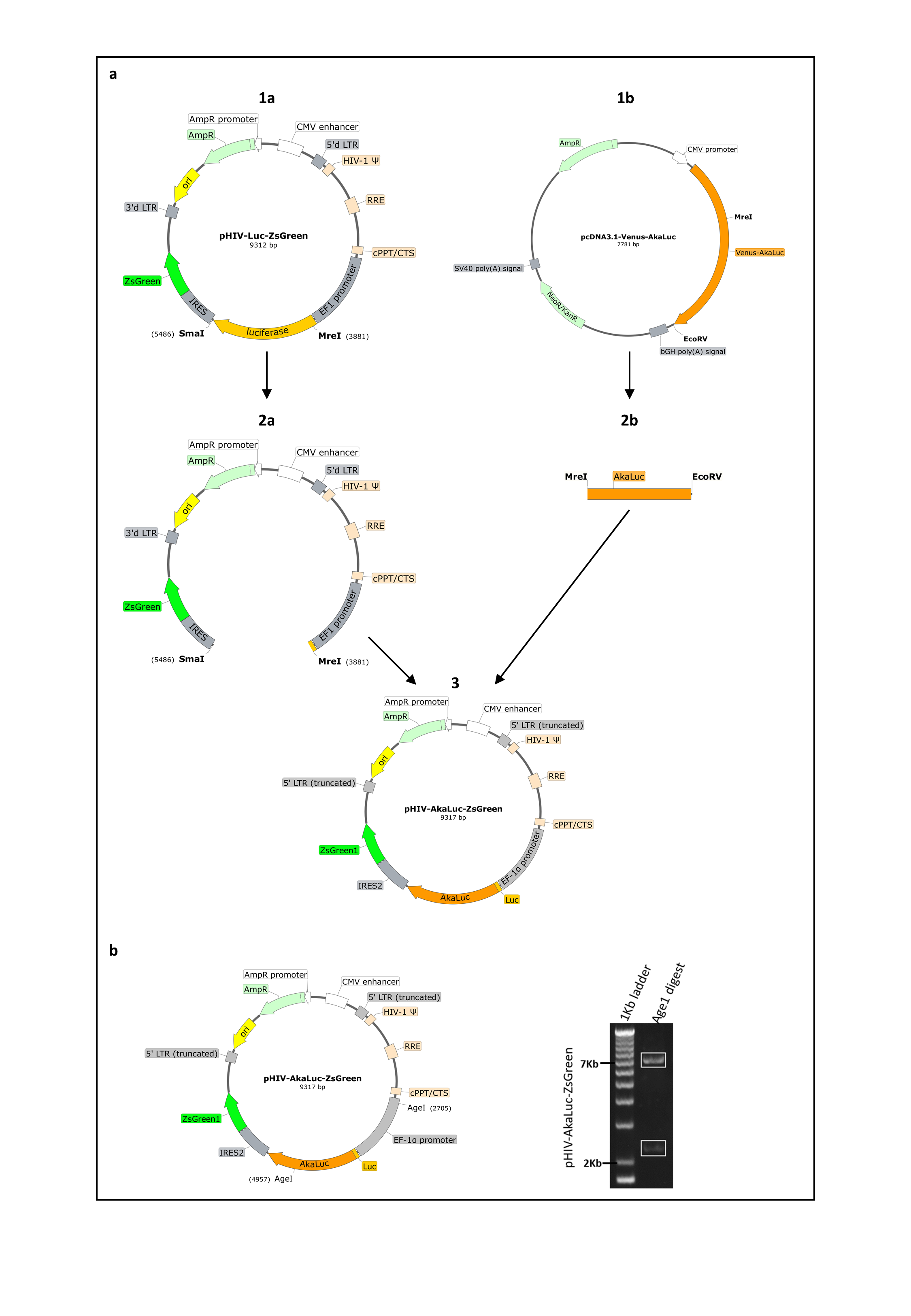


**Supplementary Figure 1| Cloning strategy to generate the pHIV-AkaLuc-ZsGreen vector. (a)** We used the pHIV-Luc2-ZsGreen vector to construct the pHIV-AkaLuc-ZsGreen vector. For that, we performed the extraction of luc2 from the pHIV-Luc2-ZsGreen (1a) plasmid using the *MreI* and *SmaI* restriction enzymes sites (2a) and the *MreI* and *EcoRV* restriction enzymes (1b) to remove the gene encoding AkaLuc from pcDNA3.1-Venus-AkaLuc (2b). The pHIV-ZsGreen backbone and AkaLuc insert were ligated together to generate the pHIV-AkaLuc-ZsGreen construct (3). **(b)** Diagnostic test digest of the pHIV-AkaLuc-ZsGreen vector using the *AgeI* enzyme sites. Gel electrophoresis shows DNA bands at ≈2.2Kb and ≈7Kb. Images produced using SnapGene software (from Insightful Science; available at snapgene.com).


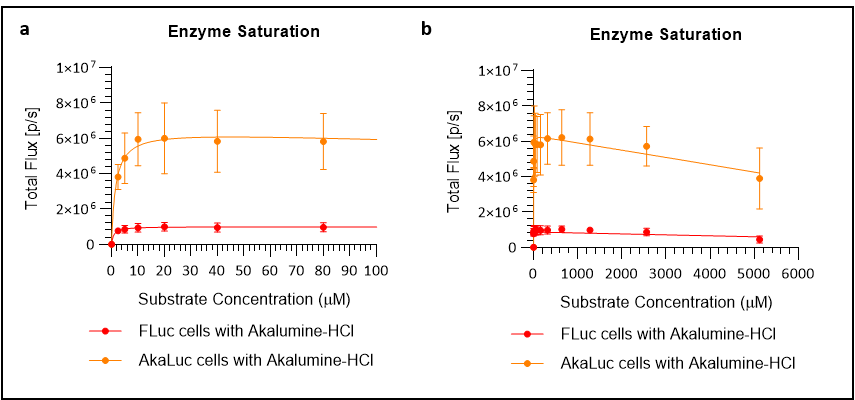


**Supplementary Figure 2| The Akalumine-HCl saturates the Akaluc reporter at low concentrations but results in a signal drop at high concentrations.** Akaluc and FLuc expressing MSCs were seeded at a density of 1.5x10^3^ cells/well and treated with rising concentrations of Akalumine-HCl (2.5 μM to 5.12 mM). **(a-b)** Light output (flux) as a function of substrate concentration, where **(a)** shows the signal obtained from 2.5 μM to 100 μM and **(b)** from 2.5 μM to 5.12 mM. Data are displayed as mean±SD from n = 3. The data was acquired using an open emission filter, a 13.3 field of view, a f-stop of 1, a binning of 8 and 10 seconds of exposure.


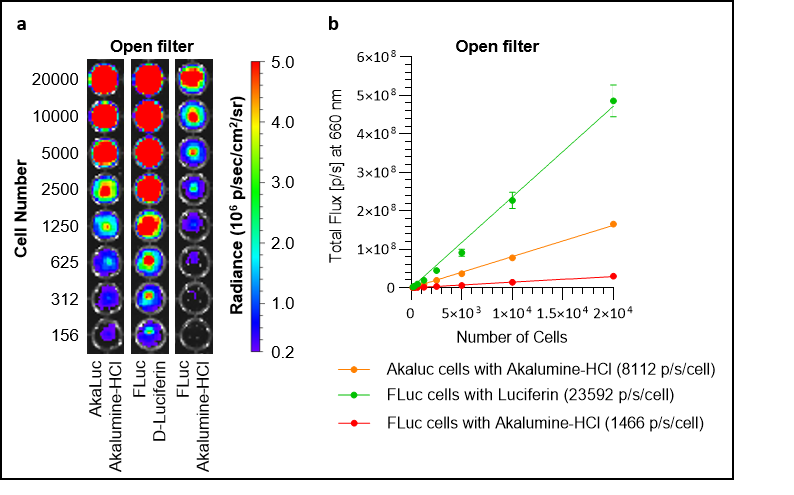


**Supplementary Figure 3| Light output as a function of cell density for the AkaLuc and FLuc reporter systems with an open emission filter.** Akaluc and FLuc expressing MSCs were seeded at density of 156 to 2x10^4^ cells/well and treated with saturating concentration of the substrates (160 μM Akalumine-HCl or 5.12 mM D-luciferin). AkaLuc expressing cells were treated with Akalumine-HCl only, whereas FLuc expressing cells were treated with AkaLuc-HCl or D-Luciferin. The signal was acquired using an open filter. **(a)** Representative BLI images of a well plate immediately after the substrate addition. **(b)** Light output (flux) as a function of cell concentration, with linear regression curves. The slope of each curve represents the flux/cell and is shown in the legend of the graph. Data are displayed as mean±SD from n=3. The signal was acquired using a 13.3 field of view, a f-stop of 1, a binning of 8 and 10 seconds of exposure.


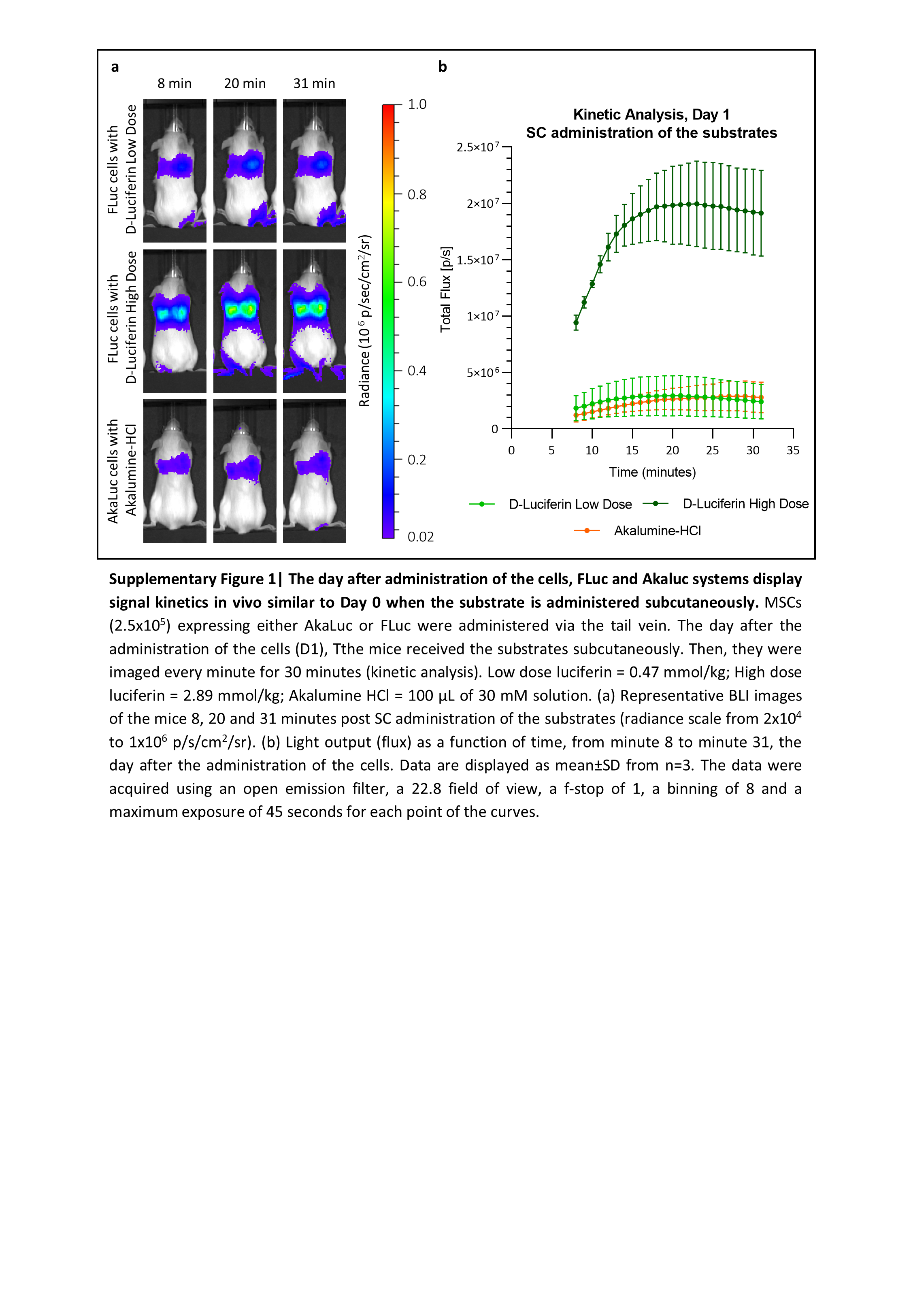


**Supplementary Figure 4| The day after administration of the cells, the FLuc and AkaLuc systems display signal kinetics *in vivo* similar to day 0, when the substrate is administered subcutaneously.** MSCs (2.5x10^5^) expressing either AkaLuc or FLuc were administered via the tail vein. The day after the administration of the cells (D1), the mice received the substrates subcutaneously. Then, they were imaged every minute for 30 minutes (kinetic analysis). Low dose D-Luciferin = 0.47 mmol/kg; High dose D-Luciferin = 2.89 mmol/kg; Akalumine‑HCl = 100 µL of 30 mM solution. **(a)** Representative images of the mice 8, 20 and 31 minutes post SC administration of the substrates (radiance scale from 2x10^4^ to 1x10^6^ p/s/cm^2^/sr). **(b)** Light output (flux) as a function of time, from minute 8 to minute 31, the day after the administration of the cells. Data are displayed as mean±SD from n=3. Acquisition parameters: no emission filter, 22.8 cm FOV, f-stop of 1, binning of 8 and a maximum exposure of 45 seconds for each time point.


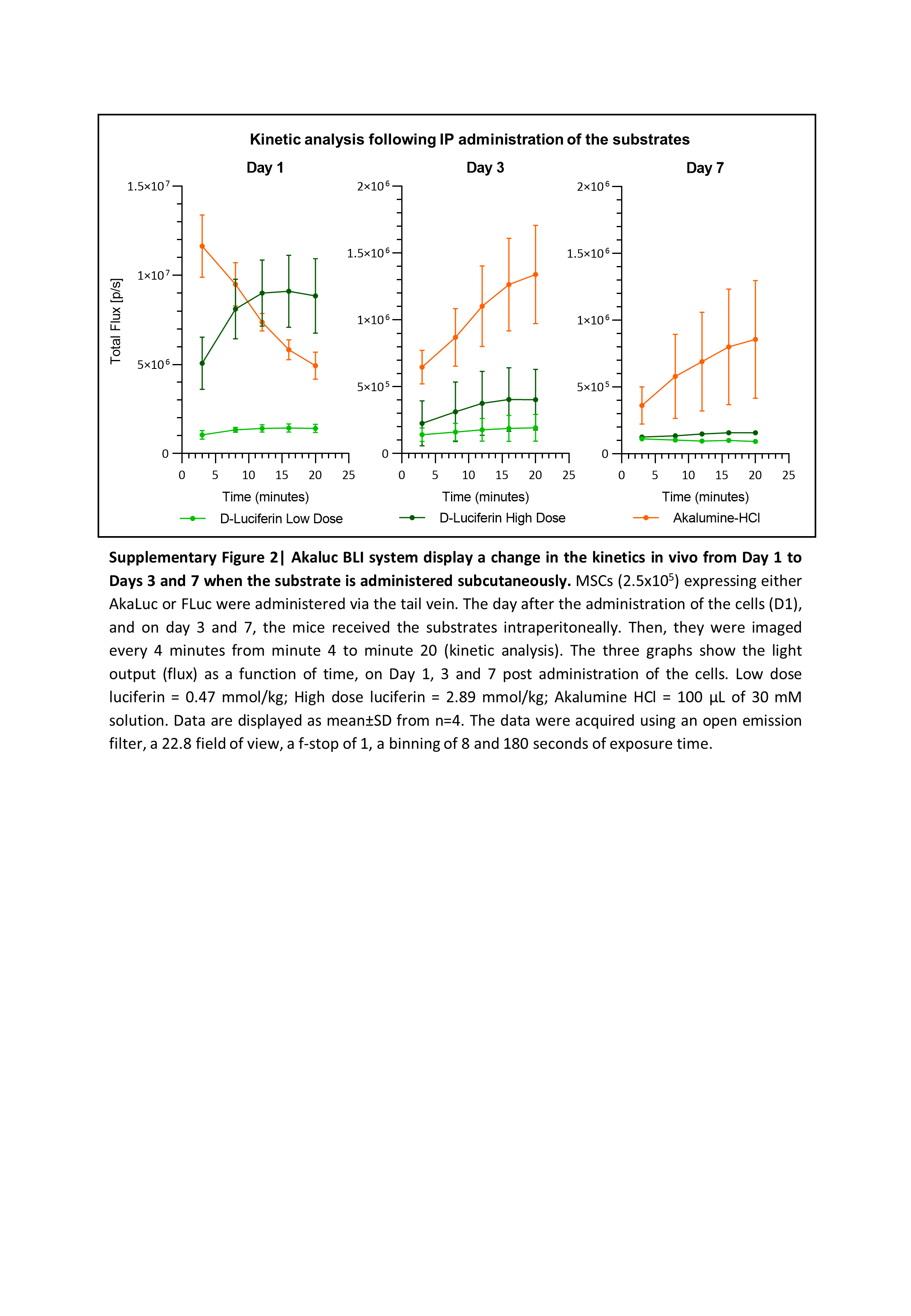


**Supplementary Figure 5| The AkaLuc system displays a change in the kinetics *in vivo* from day 1 to days 3 and 7, when the substrate is administered intraperitoneally.** MSCs (2.5x10^5^) expressing either AkaLuc or FLuc were administered via the tail vein. The day after the administration of the cells (D1), and on day 3 and 7, the mice received the substrates intraperitoneally. Then, they were imaged every 4 minutes from minute 4 to minute 20 (kinetic analysis). The three graphs show the light output (flux) as a function of time, on days 1, 3 and 7 post administration of the cells. Low dose D-Luciferin = 0.47 mmol/kg; High dose D-Luciferin = 2.89 mmol/kg; Akalumine‑HCl = 100 µL of 30 mM solution. Data are displayed as mean±SD from n=4. Acquisition parameters: no emission filter, 22.8 cm FOV, f-stop of 1, binning of 8 and 180 seconds of exposure time.

**
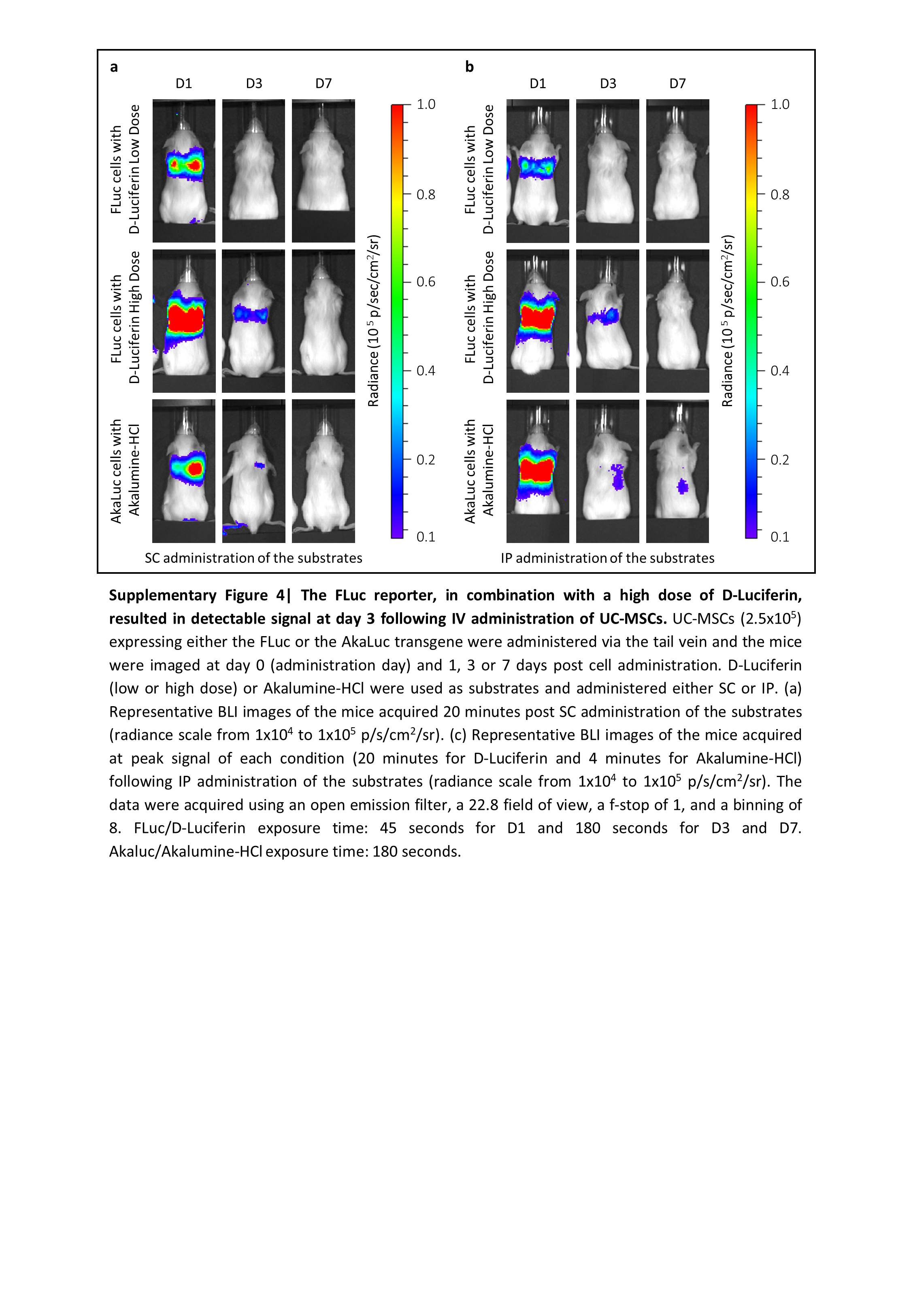
**

**Supplementary Figure 6| The FLuc reporter, in combination with a high dose of D-Luciferin, allows the detection of signal at day 3 following IV administration of UC-MSCs.** UC-MSCs (2.5x10^5^) expressing either the FLuc or the AkaLuc transgene were administered via the tail vein and the mice were imaged at day 0 (administration day) and 1, 3 or 7 days post cell administration. D-Luciferin (low or high dose) or Akalumine-HCl were used as substrates and administered either SC or IP. **(a)** Representative images of the mice acquired 20 minutes post SC administration of the substrates (radiance scale from 1x10^4^ to 1x10^5^ p/s/cm^2^/sr) on days 1, 3 and 7. **(b)** Representative images of the mice acquired at peak signal of each condition (20 minutes for D-Luciferin and 4 minutes for Akalumine-HCl) following IP administration of the substrates (radiance scale from 1x10^4^ to 1x10^5^ p/s/cm^2^/sr). Acquisition parameters: no emission filter, 22.8 cm FOV, f-stop of 1 and a binning of 8. FLuc/D-Luciferin exposure time: 45 seconds for D1 and 180 seconds for D3 and D7. AkaLuc/Akalumine-HCl exposure time: 180 seconds.


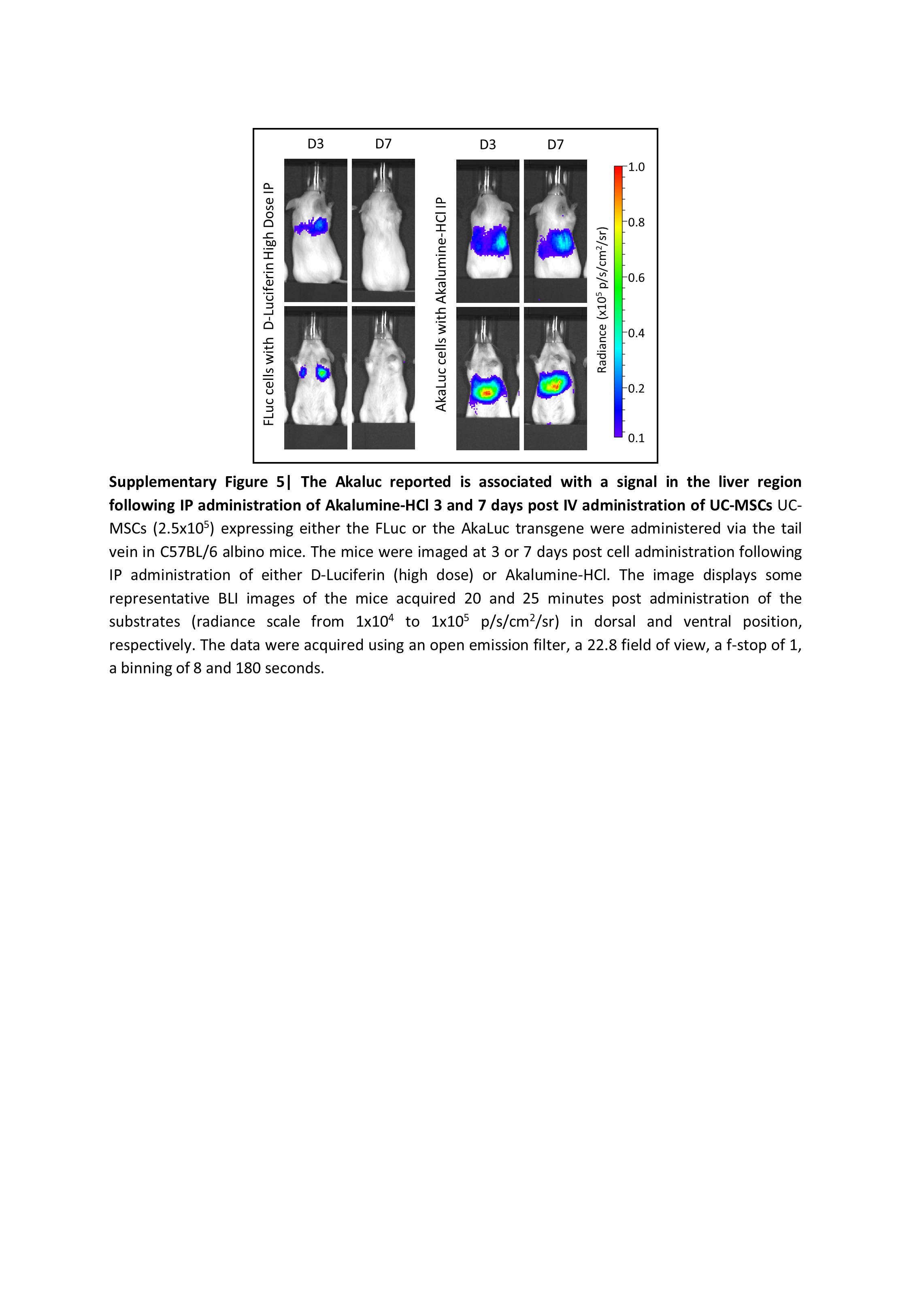


**Supplementary Figure 7| The AkaLuc reporter is associated with a signal in the region corresponding to the liver following IP administration of Akalumine-HCl at 3 and 7 days post IV administration of UC-MSCs.** UC-MSCs (2.5x10^5^) expressing either the FLuc or the AkaLuc transgene were administered via the tail vein in C57BL/6 albino mice. The mice were imaged at 3 or 7 days post cell administration, following IP administration of either D-Luciferin (high dose) or Akalumine-HCl. The image displays representative images of the mice acquired approximately 20 minutes post administration of the substrates (radiance scale from 1x10^4^ to 1x10^5^ p/s/cm2/sr) in dorsal and ventral position, respectively. Acquisition parameters: no emission filter, 22.8 cm FOV, f-stop of 1, binning of 8 and 180 seconds exposure.

**
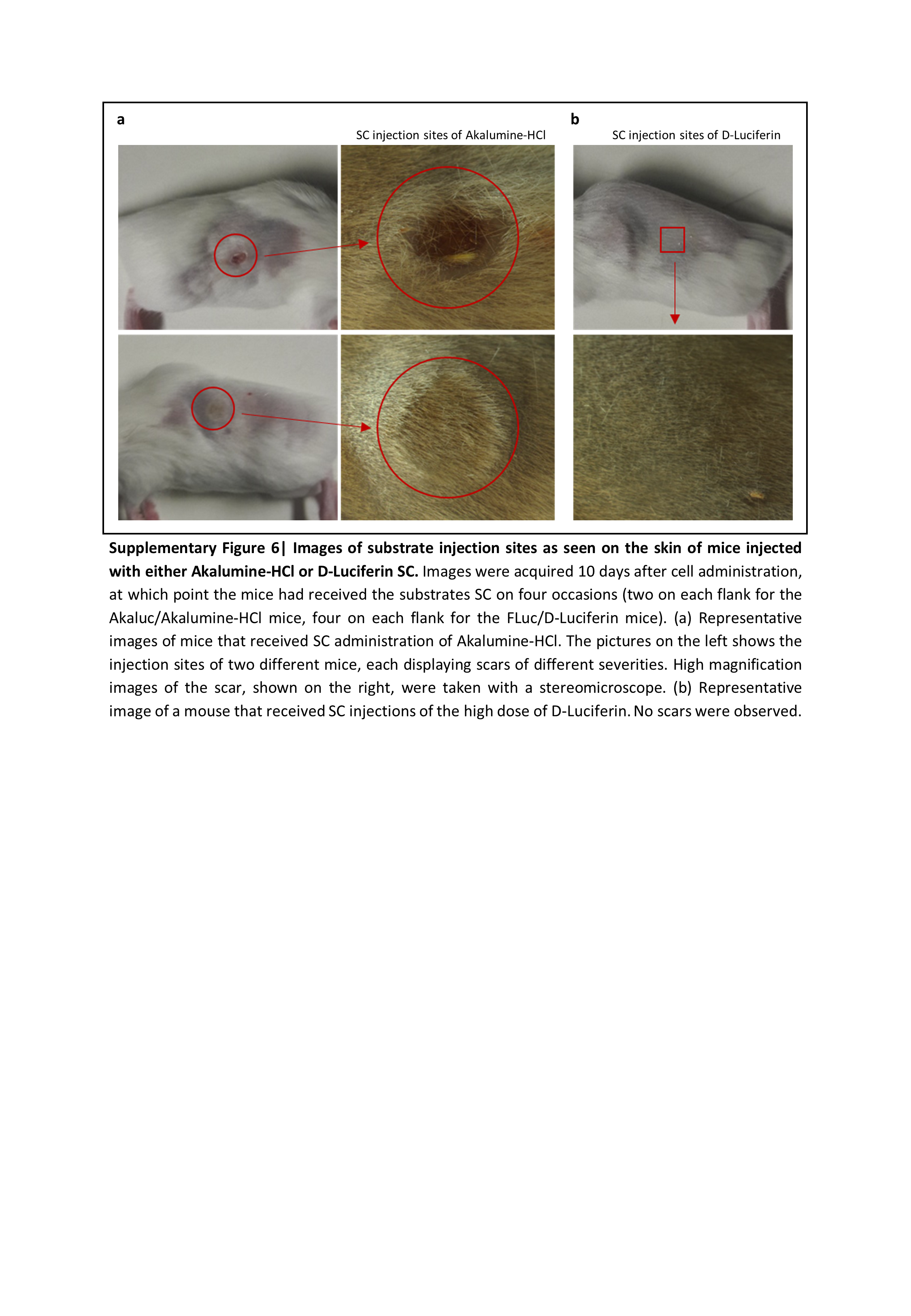
**

**Supplementary Figure 8| Images of substrate injection sites as seen on the skin of mice injected with either Akalumine-HCl or D-Luciferin SC.** Images were acquired 10 days after cell administration, at which point the mice had received the substrates SC on four occasions (two on each flank for the AkaLuc/Akalumine-HCl mice, four on each flank for the FLuc/D-Luciferin mice). **(a)** Representative images of mice that received SC administration of Akalumine‑HCl. The pictures on the left shows the injection sites of two different mice, each displaying lesions of different severities. High magnification images of the lesion, shown on the right, were taken with a stereomicroscope. **(b)** Representative image of a mouse that received SC injections of the high dose of D-Luciferin. No lesions were observed.

**Supplementary Table 1:** experimental set up to evaluate the signal kinetics of the two BLI systems. This table refers to the data presented in Figure 4, Supplementary Figure 2 and 3.

| Cells Injected IV | N°  of cells injected | Substrate used | Substrate concentration (mM) | Volume of substrate administered (μL/g of body weight) | N° of sites of substrate injection | N° of animals^[[1]](#footnote-2)^ |
| --- | --- | --- | --- | --- | --- | --- |
| AkaLuc | 2.5x10^5^ | Akalumine-HCl (SC) | 30 | Always a total volume of 100 μL | 1 | 3 |
| AkaLuc | 2.5x10^5^ | Akalumine-HCl (IP) | 30 | Always a total volume of 100 μL | 1 | 4 |
| FLuc | 2.5x10^5^ | D-Luciferin (SC) | 47 | 10 | 1 | 3 |
| FLuc | 2.5x10^5^ | D-Luciferin (IP) | 47 | 10 | 1 | 4 |
| FLuc | 2.5x10^5^ | D-Luciferin (SC) | 144.5 | 20 | 2 | 3 |
| FLuc | 2.5x10^5^ | D-Luciferin (IP) | 144.5 | 20 | 1 | 4 |

**Supplementary Table 2:** experimental set up to evaluate the signal following SC or IP administration of the substrates. This table refers to the data presented in Figure 5 and Supplementary Figure 4 and 5.

| Cells Injected IV | N°  of cells  injected | Substrate used | Substrate  concentration (mM) | Volume of substrate administered (μL/g of body weight) | N° of sites of substrate injection | N° of animals^1^ |
| --- | --- | --- | --- | --- | --- | --- |
| AkaLuc | 2.5x10^5^ | Akalumine-HCl (SC) | 30 | Always a total volume of 100 μL | 1 | 7 |
| AkaLuc | 2.5x10^5^ | Akalumine-HCl (IP) | 30 | Always a total volume of 100 μL | 1 | 4 |
| FLuc | 2.5x10^5^ | D-Luciferin (SC) | 47 | 10 | 1 | 3 |
| FLuc | 2.5x10^5^ | D-Luciferin (IP) | 47 | 10 | 1 | 4 |
| FLuc | 2.5x10^5^ | D-Luciferin (SC) | 144.5 | 20 | 2 | 7 |
| FLuc | 2.5x10^5^ | D-Luciferin (IP) | 144.5 | 20 | 1 | 4 |

**Supplementary Table 3:** experimental set up to evaluate the unspecific signal of D-Luciferin and Akalumine‑HCl. This table refers to the data presented in Figure 6.

| Substrate used | Concentration (mM) | Volume administered (μL/g of body weight) | Sites of administration | N° of animals^1^ |
| --- | --- | --- | --- | --- |
| Akalumine-HCl | 30 | Always a total volume of 100 μL | IP (1 site) | 3 |
| D-Luciferin | 144.5 | 20 | IP (1 site) | 3 |
| D-Luciferin | 144.5 | 20 | SC (2 sites) | 3 |

1. The sample size in all experimental groups was at least 4, with animals which were misinjected with cells (i.e., did not receive the full 2.5x10^5^ dose) excluded from the analysis. In our experience a n=3 is the minimum for adequate power in such studies. [↑](#footnote-ref-2)
